# Understanding and improving compound pressures in general practice: a realist review protocol

**DOI:** 10.3399/BJGPO.2025.0073

**Published:** 2026-02-25

**Authors:** Emily Owen-Boukra, Ruth Abrams, Tanya Cohen, Claire Goodman, Cecily Henry, Laura Ingle, Kamal Mahtani, Margaret Ogden, Nia Wyn Roberts, Rupesh Shah, James Thomas, Geoff Wong, Sophie Park

**Affiliations:** 1 Nuffield Department of Primary Care Health Sciences, University of Oxford, Oxford, UK; 2 School of Health Sciences, University of Surrey, Surrey, UK; 3 Centre for Research in Public Health and Community Care, University of Hertfordshire, Hatfield, UK; 4 Bodleian Health Care Libraries, University of Oxford, Oxford, UK; 5 School of Engineering and Innovation, The Open University, Milton Keynes, UK; 6 UCL Social Research Institute, University College London, London, UK, UK

**Keywords:** compound pressures, general practice, realist review, patient care, primary health care

## Abstract

**Background:**

Compound pressures (CP) impact the role of general practice in supporting human health. These pressures include climate change, pandemics, and financial crises. CP can be predictable, pre-determined, or unpredictable in nature and scope. Strategies to address the demands arising from CP range from short-term initiatives to buffering existing general practice systems to ensure flexible and agile resources. Interventions designed to prevent, identify, and manage CP may result in both intended and unintended outcomes.

**Aim:**

To conduct a realist appreciative inquiry, realist review, and three embedded studies within a review (SWAR) about CP affecting general practice and the delivery of effective, equitable patient care.

**Design & setting:**

Realist appreciative inquiry, realist review, and three SWARs.

**Method:**

We will conduct a realist appreciative inquiry facilitating patient and stakeholder input into the review scope, focus, and initial programme theory (IPT) development. This approach emphasises the identification of assets, successes, hopes, and aspirations to enable positive change. Based on these insights, we will conduct a realist review of empirical and grey literature. This project includes three elements of methodological innovation (SWARs). First, evaluation of how appreciative inquiry can inform IPT development. Two further SWARs will examine how artificial intelligence might (a) support the identification of relevant resources at title and abstract, and full-text stages; and (b) support data extraction and analysis in future realist reviews.

**Conclusion:**

Our research aims to understand the effects of CP on general practice, supporting preparation and solutions that can inform future policies, interventions, and support systems.

## How this fits in

The term, compound pressures, as defined by the National Institute for Health and Care Research (NIHR), refers to an increasingly complex set of challenges facing health and social care. CP have implications for patients, caregivers, healthcare professionals, and systems such as general practice. Our research will encompass a realist appreciative inquiry, realist review, and three methodological studies within a review (SWARs) to examine the characteristics of CP in general practice, proposed solutions, and both the intended and unintended outcomes of CP interventions, with a particular focus on maximising effective and equitable patient care. We will address existing knowledge gaps and establish a foundation for more targeted approaches and strategies to mitigate the effects of CP.

## Introduction

Compound pressures (CP) on the NHS and social care systems have become an important concern for patients, healthcare professionals, and policymakers. 'The NIHR refer to compound pressures as an increasingly complex set of challenges facing health and social care.^1^ These pressures include, but are not limited to, winter pressures, climate change, weather-related events (for example, global warming, flooding, cold, or heat), financial crises, and pandemics (for example, COVID-19). CP are anticipated to persist and escalate in both frequency and severity owing to global challenges, such as climate change and international conflicts, which impact food security, energy, housing, and financial stability. The capacity to respond to these crises is interconnected with factors such as the cost of living, social deprivation, population morbidity (levels of disease and ill health in the community), and the resources available within health and social care systems.^2^ The Health and Social Care Committee report on the future of general practice indicated that 90% of health care is delivered in primary care.^3^ Consequently, it is crucial to enhance our understanding of how CP can impact general practice and its role(s), contribution, and collaboration with the broader health and social care system. This includes the impact of CP interventions on patient equity, workforce composition and wellbeing, as well as the relationship between the workforce and organisational structures.

This research addresses these critical concerns using realist appreciative inquiry, realist review, and studies within a review (SWAR) methodology. The realist approach enables us to examine how and under what circumstances interventions and initiatives may or may not work, and to form explanations for these observed similarities and differences.^4,5^ This approach is particularly suitable for this research, which aims to enhance understanding of the nature of CP and identify appropriate future mitigation and support strategies. Additional methodological details are provided in our PROSPERO registration (CRD42025636290).^6^ The scope and focus of this review will be informed by preliminary patient and public involvement (PPI) and stakeholder engagement, using an appreciative inquiry approach to explore not only inherent challenges but also positive aspects, successful outcomes, and mitigation strategies. In addition, the project incorporates three SWAR components^7^ to inform the methodology for future realist reviews.

## Method

### Aims and objectives

Aims: To conduct a realist appreciative inquiry, realist review, and three embedded SWARs about CP affecting general practice and the delivery of effective and equitable patient care.

Research questions: How, why, when, and in what circumstances might CP be addressed in general practice to maximise effective and equitable patient care?

Specifically, within the available literature:

1. What are the characteristics of CP in UK general practice?
2. What are the reported general practice solutions to CP?
3. What are the intended and unintended outcomes of CP interventions for general practice?
4. How can general practice support approaches to CP?
5. How can patients and the public work with services to approach CP in general practice?

In addition, three SWARs ask:

1. What is the value of an appreciative inquiry approach in informing the initial programme theory (IPT) development for a realist review?
2. How might artificial intelligence (AI) support (or not) the identification of relevant resources at title and abstract and full-text stages?
3. How can AI be used (or not) to support data extraction and analysis in future realist reviews?

### Research plan and methods

We will begin by conducting a realist appreciative inquiry, which is a form of participatory action research.^8^ This approach comprises a strengths-based framework designed to facilitate positive change by identifying and enhancing individuals’ assets, successes, hopes, and dreams.^8,9^ This framework shifts away from traditional problem-focused orientations by instead seeking to identify: ‘what is working; why is it working; and what could be in the future?’. The appreciative inquiry will inform the initial search terms and focus for our realist review, such as the characteristics we initially focus on and the potential interventions and solutions. Subsequently, a realist review will synthesise data from the available literature to produce and refine an evidence-based programme theory about causal explanations in relation to CP. This will support: (1) the delivery of effective and equitable patient care; and (2) the inter-relations between CP challenges and solutions with general practice service provision.

### Research design and theoretical framework

A realist approach is ideal for exploring this complex and new field of literature. We will include grey literature, such as recent policy changes, linking this with elements of PPI and stakeholder theories and substantive published evidence.^4^ From the outset, a realist approach acknowledges that different contexts lead to different outcomes, thus, identifying the underlying generative mechanisms regarding how responses to CP work (or not). The project findings will be used to identify important system-level contexts that may be amenable to change.^4^ Our patient and stakeholder involvement work encompasses the following:

1. **IPT development:** We will read relevant literature to develop an IPT. The IPT will: (1) explore the characteristics of CP in UK general practice; (2) examine how general practice may support CP approaches; and (3) explore how patients may collaborate with general practice services to address CP. We will expand this IPT in collaboration with our PPI and stakeholder groups.
2. **Discover:** Through structured group discussions and narrative-based inquiry, stakeholders will articulate their strengths, virtues, interests, and experiences of collaborative success. They will share instances of approaching and addressing CP, where they experienced high levels of engagement and motivation. PPI participants will also share experiences of successful CP interventions and responses, and the positive impact on their care.
3. **Dream:** This phase will include PPI participants and stakeholders envisioning potential enhancements to patient care; exploring strengths, attributes, and virtues; and examining how these could be synthesised, leveraged, and combined. Through a series of creative activities and structured tasks, PPI participants and stakeholders will engage in discussions regarding their preferred approaches to addressing and resolving CP. Additionally, PPI participants will describe their desired approach for collaborative efforts among key stakeholders in responding to CP.

Through structured group discussions and narrative-based inquiry, stakeholders will articulate their strengths, virtues, interests, and experiences of collaborative success. They will share instances of approaching and addressing CP, where they experienced high levels of engagement and motivation. PPI participants will also share experiences of successful CP interventions and responses, and the positive impact on their care.

Our realist review protocol will build on the realist appreciative inquiry and will be guided by Pawson’s five iterative stages^4^ and current quality and publication standards (Realist And Meta-narrative Evidence Syntheses: Evolving Standards; RAMESES),^10,11^ which are outlined below.

**Step 1:** *Locate existing theories:* Following the preliminary work conducted during the appreciative inquiry phase, we will expand our IPT for the realist review. This will describe assumptions regarding important influences on CP. Where possible, it will outline potentially important contexts, mechanisms, and outcomes pertaining to CP. We will invite our stakeholder and PPI groups to provide feedback and examine the differences, dissonance, and commonalities between the appreciative inquiry initial phase and what we are identifying within the initial review searches and identified literature.

**Step 2:** *Search for evidence*: We will conduct systematic searches to identify a relevant ‘body of literature’ with which to refine the IPT from Step 1. The search strategy will be designed, piloted, and conducted by NR. It is anticipated that our searches will combine free text and subject heading (for example, MeSH) terms for CP and general practice, respectively. The following databases are expected to be searched: MEDLINE; Embase; CINAHL; PsycInfo; Cochrane Library; HMIC; the Web of Science (Science and Social Science Citation Indexes); and web sources including Overton and Google Scholar. Additional relevant databases will be searched, and ‘cited by’ article searches and searches of citations contained in the reference lists of documents will be undertaken. Grey literature will be identified through a search of relevant websites.

**Step 3:** *Article selection:* This process comprises three stages: screening based on title and abstract; screening based on full text; and final selection of full-text documents based on their relevance (whether they contain data that contribute to theory building and/or testing) and rigour (whether the methods used to generate the relevant data are credible and trustworthy).^4^ To ensure consistency, a random 10% sample of decisions will be independently checked at each stage by RA. Any discrepancies will be addressed through discussion with the research team.

**Step 4:** *Extracting and organising data:* Data extraction and organisation will be undertaken by EOB. Discrepancies and disagreements will be discussed with the research team. The included full texts will be uploaded into EPPI-Reviewer for coding. These will be both coded inductively (codes created to categorise data reported in included studies), deductively (codes created in advance of data extraction and analysis, as informed by the IPT), and retroductively. Each new element of relevant data will be used to refine the programme theory, and as it is refined, included studies will be re-scrutinised to search for relevant data that may have been missed initially. A random sample of 10% extracted data and coding will be independently checked for quality control.

**Step 5:** *Synthesising the evidence and drawing conclusions:* Data analysis will use a realist logic of analysis to make sense of the IPT. EOB will undertake this step with support from the research team and PPI group and stakeholders. We will use interpretive cross-case comparison to explain how and why observed outcomes have occurred, for instance, by comparing literature in which interventions have produced successful (or unsuccessful) outcomes to CP, examining how context has influenced reported findings. We will use a proven analysis and synthesis process that we have successfully used for previous large-scale funded realist reviews.^12^

**Step 6:** Two co-production events will build on the PPI group’s and stakeholders’ appreciative inquiry stages and the realist review. Participants will be invited to reflect on the project findings regarding both CP and the three SWAR approaches. We will extend the appreciative inquiry approach in these final discussions to co-produce recommendations with PPI and stakeholders concerning CP (for example, priorities, key interventions, and approaches) and future realist review methods (including the value of appreciative inquiry and AI).

This review incorporates three methodological studies:

- SWAR 1 examines how appreciative inquiry can inform IPT development;
- SWAR 2 explores how AI can assist with identification of key relevant papers; and
- SWAR 3 uses AI alongside humans for data extraction and analysis.

### Dissemination and impact

Two events will be conducted with relevant public, practitioner, and policy participants to co-produce resources and recommendations regarding (a) future CP strategies and interventions, and (b) how SWARs can inform future realist methods. These events will inform the development of outputs (for example, short reports, academic papers) tailored for policy, practitioner, and public audiences.

## Discussion

### Summary

By conducting a realist appreciative inquiry, realist review, and three methodological SWARs, we will generate and refine an evidence-based programme theory regarding causal explanations in relation to CP to support: (1) the delivery of effective and equitable patient care; and (2) the inter-relations between CP challenges and solutions with general practice service provision. Our research will provide nuanced insights regarding reported solutions to CP, the intended and unintended outcomes for general practice to CP interventions, and how patients can collaborate with services to approach CP in general practice to maximise effective and equitable patient care. We will address existing knowledge gaps and provide a foundation for more targeted approaches to mitigate the effects of CP.

### Strengths and limitations

Our realist appreciative inquiry and review are well-suited to this novel and expanding field of knowledge. Realist analysis can address the diverse contexts in which various mechanisms and outcomes emerge. The realist approach also facilitates the inclusion of PPI group and stakeholder perspectives, thereby informing selection, analysis, and dissemination approaches throughout the project timeline. This is crucial to ensure that selected texts and analytical categories are pertinent to general practice and relevant to contemporary challenges and potential practice and policy solutions. Our research also builds on a previous realist review that examined the sustainability of the general practice workforce.^13–15^ However, a potential limitation of this research is its reliance on existing literature. As CP is a new concept, the literature surrounding it may be limited. Nonetheless, it is anticipated that many ‘older’ issues may reasonably be re-conceptualised as CP, potentially providing relevant data.

### Implications for research and practice

By identifying the interactions between contextual factors and underlying mechanisms, this research has the potential to produce transferable causal explanations that can inform the development of targeted interventions, policies, and support strategies. The findings may facilitate the formulation of more flexible and responsive approaches for managing CP in general practice. Additionally, the three innovative methodological SWARs will support efforts to maximise opportunities for evidence-informed policy and practice, while ensuring deliberate and high quality engagement with relevant literature. These SWARs will serve as a foundation for future realist research approaches.

## Trial registration number

PROSPERO registration (CRD42025636290)

## Data

N/A
